# Perfusion-Based Bioreactor Culture and Isothermal Microcalorimetry for Preclinical Drug Testing with the Carbonic Anhydrase Inhibitor SLC-0111 in Patient-Derived Neuroblastoma

**DOI:** 10.3390/ijms23063128

**Published:** 2022-03-14

**Authors:** Zihe Huo, Remo Bilang, Claudiu T. Supuran, Nicolas von der Weid, Elisabeth Bruder, Stefan Holland-Cunz, Ivan Martin, Manuele G. Muraro, Stephanie J. Gros

**Affiliations:** 1 Department of Pediatric Surgery, University Children’s Hospital Basel, 4031 Basel, Switzerland; zihe.huo@ukbb.ch (Z.H.); remo.bilang@ukbb.ch (R.B.); stefan.holland-cunz@ukbb.ch (S.H.-C.); 2 Department of Clinical Research, University of Basel, 4031 Basel, Switzerland; nicolas.vonderweid@ukbb.ch; 3 Department Neurofarba, Sezione di Scienze Farmaceutiche, University of Florence, 50121 Florence, Italy; claudiu.supuran@unifi.it; 4 Department of Hematology and Oncology, University Children’s Hospital Basel, 4031 Basel, Switzerland; 5 Institute of Pathology, University Hospital Basel, 4031 Basel, Switzerland; elisabeth.bruder@usb.ch; 6 Tissue Engineering, Department of Biomedicine, University of Basel and University Hospital Basel, 4031 Basel, Switzerland; ivan.martin@unibas.ch (I.M.); manuele.muraro@unibas.ch (M.G.M.)

**Keywords:** carbonic anhydrase IX, carbonic anhydrase XII, SLC-0111, 3D culture, organotypic slice culture, hypoxia, novel inhibitor, neuroblastoma, bioreactor, preclinical drug screening, isothermal microcalorimetry for drug assessment

## Abstract

Neuroblastoma is a rare disease. Rare are also the possibilities to test new therapeutic options for neuroblastoma in clinical trials. Despite the constant need to improve therapy and outcomes for patients with advanced neuroblastoma, clinical trials currently only allow for testing few substances in even fewer patients. This increases the need to improve and advance preclinical models for neuroblastoma to preselect favorable candidates for novel therapeutics. Here we propose the use of a new patient-derived 3D slice-culture perfusion-based 3D model in combination with rapid treatment evaluation using isothermal microcalorimetry exemplified with treatment with the novel carbonic anhydrase IX and XII (CAIX/CAXII) inhibitor SLC-0111. Patient samples showed a CAIX expression of 18% and a CAXII expression of 30%. Corresponding with their respective CAIX expression patterns, the viability of SH-EP cells was significantly reduced upon treatment with SLC-0111, while LAN1 cells were not affected. The inhibitory effect on SH-SY5Y cells was dependent on the induction of CAIX expression under hypoxia. These findings corresponded to thermogenesis of the cells. Patient-derived organotypic slice cultures were treated with SLC-0111, which was highly effective despite heterogeneity of CAIX/CAXII expression. Thermogenesis, in congruence with the findings of the histological observations, was significantly reduced in SLC-0111-treated samples. In order to extend the evaluation time, we established a perfusion-based approach for neuroblastoma tissue in a 3D perfusion-based bioreactor system. Using this system, excellent tissue quality with intact tumor cells and stromal structure in neuroblastoma tumors can be maintained for 7 days. The system was successfully used for consecutive drug response monitoring with isothermal microcalorimetry. The described approach for drug testing, relying on an advanced 3D culture system combined with a rapid and highly sensitive metabolic assessment, can facilitate development of personalized treatment strategies for neuroblastoma.

## 1. Introduction

Neuroblastoma arises from the sympathetic nervous system and is the most common solid tumor diagnosed in the first year of life [1]. With an incidence of 1:7000 to 1:8000, neuroblastoma is the most common extracranial solid malignancy in children [2,3]. Neuroblastoma is a heterogenous malignancy with a wide spectrum of cellular subtypes ranging from epithelial-like tissues to neuronal tissues with a wide variety of clinical manifestations. Although modern treatment strategies have led to increased survival rates, neuroblastoma still contributes to 15% of child deaths from cancer, and high-risk patients still face poor outcomes [2,3]. Characteristically, clinical behaviors and clinical outcomes are highly heterogeneous. The reasons why tumors spontaneously regress or progress to multi-organ manifestation and chemoresistance is largely undetermined [4,5,6]. Despite recent advances through clinical studies, almost 80% of patients with advanced disease do not show sufficient response to treatment [7,8].

First, explanations for inter- and intra-tumor heterogeneity could be found in the manifestation of genetic, segmental aberrations, such as loss of chromosomes 1p, 3p, 4p, 11q or gain of chromosomes 1q, 2p, 17q [3]. Furthermore, several promoter genes including their core regulatory circuitries (CRCs) have been identified for neuroblastoma, such as MYCN, ALK and TERT [9]. These factors can in themselves complicate effective targeted therapy, but fail to fully explain biological and clinical heterogeneity. We are still lacking essential understanding of neuroblastoma biology, including local progression and metastatic spread, failure of drug response, and oftentimes severe and late manifestations of side effects in survivors [10].

Neuroblastoma is classified as a rare disease. High-risk neuroblastoma patients are included in the High-Risk Neuroblastoma Study 1.8 of SIOP-Europe (SIOPEN) trial. Currently, clinical trials allow testing of experimental therapies only in very high-risk patients at very advanced stages of tumor disease. This disease being rare, tumor biopsy material at times of diagnosis is sparse, which presents an even greater challenge for introducing new therapeutic drugs. At the same time, a major challenge in the development of new therapeutics is the great disparity between preclinical studies and in vivo trials [11]. Two-dimensional (2D) cell culture models allow only limited investigation of cell-cell interaction. Three-dimensional (3D) mono cell constructs are missing important components of the tumor microenvironment, such as the structural tumor architecture, metabolic changes and immune responses. Animal models are restricted by the failure to represent the specific pediatric, immature immune microenvironments of developing children and their tumors.

Using tumor slice culture in a perfusion-based culture system could be an important alternative as it maintains an intact tumor microenvironment by forcing the media through the tissue [12,13] while overcoming typical limitations of static cultures, including limited mass transport, i.e., nutrient delivery and waste removal, particularly in a central part of the scaffold and tissue construct [12]. Formerly, perfusion-based cell cultures have demonstrated a unique ability to generate tissue constructs displaying biological and structural characteristics comparable with those of primary tissues [13,14]. More recently, the perfusion bioreactor has proved helpful for a mid-term culture of human breast and colorectal cancer tissues [15,16].

The final but most important challenge is screening of drug efficacy in representative preclinical studies. Two-dimensional cultures can be assessed by viability or proliferation assays and animal models by several imaging techniques; high-throughput screening (HTS) addresses this challenge by using a patient-based cancer cell isolation approach [17,18]. The challenge of achieving fast, precise and reproducible therapeutic screening of intact patient-derived tissue remains unsolved. A possible solution may be found in using isothermal microcalorimetry for rapid tumor response assessment [19].

Cancer cells are characterized by dysregulated cell proliferation, and the blood vessels that form within solid tumors are often structurally and functionally abnormal, resulting in severe hypoxia [20]. Adaptation of cancer cells to the hypoxic microenvironment is regulated through physiological responses to hypoxia that are mediated by hypoxia-inducible factors, namely HIF-1α and HIF-2α. As a result of these, hypoxic cancer cells can acquire enhanced invasive and metastatic properties in addition to resistance to chemotherapy and radiation therapy; these together constitute the lethal cancer phenotype [20,21]. The HIF-1α-dependent enzyme carbonic anhydrase IX (CAIX), in addition to its isoform carbonic anhydrase XII (CAXII), amongst others, have been shown to be involved in numerous pathological processes, including tumorgenicity, tumor cell invasion, tumor progression and poorer survival in several other solid tumors [22,23]. We have previously shown a poorer prognosis for CAIX-positive neuroblastoma patients in addition to the influence of hypoxia on progression and metastases in this disease, and were successfully able to inhibit neuroblastoma cell growth in vivo by applying the experimental CAIX inhibitors FC5-207A and FC8-325A [24,25]. We have identified further hypoxia-associated prognostic factors for neuroblastoma including CXCR4, PGK1 and AQP1 leading to increased metastases [26,27,28]. Especially, AQP1 furthers tumor cell migration and acts independently of other known adverse factors, such as NMYC or NCAM [27,28].

In our current study we exploit the advantage of using a 3D model of intact neuroblastoma slice culture in combination with advanced tissue engineering techniques, such as integration of the tumor into a perfusion-based bioreactor while maintaining its essential structure and microenvironment. Monitoring of treatment response to the CAIX/CAXII inhibitor SLC-0111, which currently is in phase Ib/II clinical trials for adult solid cancers [29], is performed with isothermal microcalorimetry of the intact tumor tissue. This allows us to monitor responses to the experimental treatment of each patient’s specific tumor.

## 2. Results

### 2.1. Expression of CAIX and CAXII in Neuroblastoma and Adrenal Gland

**Figure 1:**
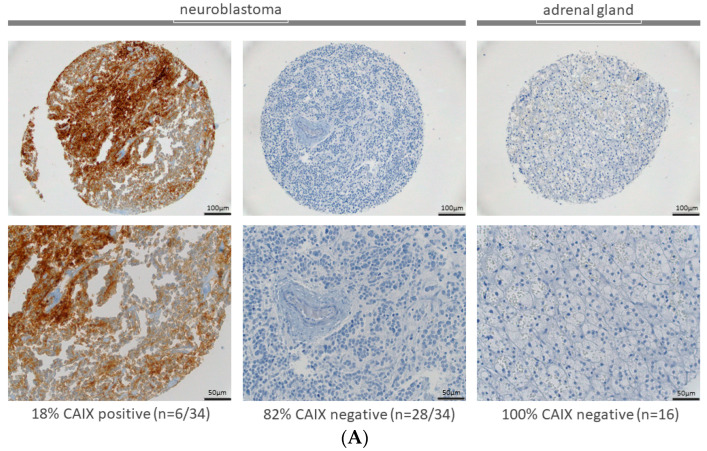

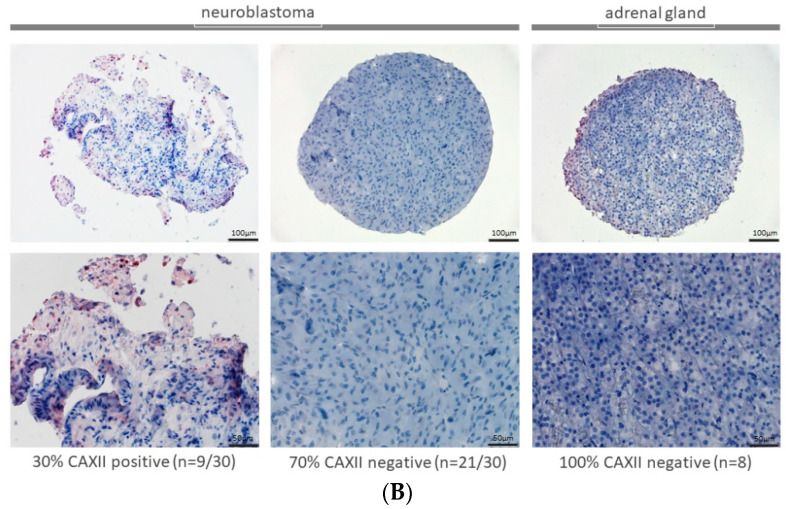
(**A**,**B**) Expressions of CAIX and CAXII in neuroblastoma. CAIX expression was observed in 18% (n = 6/34) and CAXII in 30% (n = 9/30) of neuroblastoma samples of TMA. Representative images of positive and negative CAIX expressions are shown in the first two columns. Normal adrenal gland tissue was negative for CAIX and CAXII in 100% of the samples (third) column.

Thirty-four neuroblastoma patients’ samples in addition to 16 normal adrenal gland tissues were available on a TMA at the Institute of Pathology at the University Hospital Basel, and revealed a positive CAIX expression of 18% (n = 6/34) in neuroblastoma samples. All samples with healthy adrenal gland tissue were negative (n = 16) for CAIX expression. Representative examples for CAIX staining of the TMA are shown in Figure 1A. CAXII was positive in 30% of neuroblastoma samples on the TMA (n = 9/30). All samples with healthy adrenal gland tissue were negative (n = 8) for CAXII expression. Representative examples for CAXII staining of the TMA are shown in Figure 1B.

### 2.2. Expression of CAIX and CAXII in Neuroblastoma Cells in 2D Cultures

**Figure 2:**
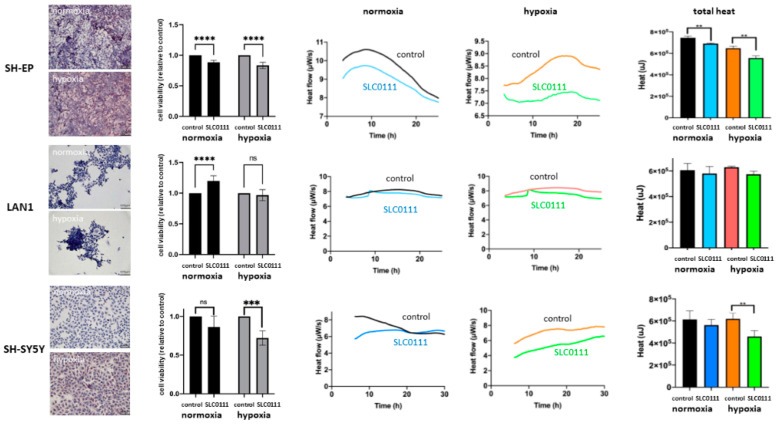
Inhibition with SLC-0111 in 2D cultures. Inhibition of neuroblastoma cell lines SH-EP, LAN1 and SH-SY5Y with SLC0111 are depicted. First column: CAIX expression of cells under hypoxic and normoxic conditions. Second column: cell viability after treatment relative to control (100%) under normoxic and hypoxic conditions (ns not significant, *** *p* < 0.001, **** *p* < 0.0001). Third/fourth columns: heat flow over time (µW/s) of treated and untreated cells under normoxia and hypoxia as measured by microcalorimetry. Fifth column: Total heat development (µJ) during observation period (corresponding to heat flow over time) measured by microcalorimetry. First row: SH-EP cell lines, that express CAIX under normoxia and even more strongly under hypoxia, react to treatment with SLC-0111 with a reduction in cell viability. At the same time, significantly reduced thermogenesis (** *p* < 0.01) can be measured by microcalorimetry. Second row: LAN1 cells that do not express CAIX to a relevant extent do not significantly react to the inhibitor in either assay. Third row: SH-SY5Y cells react significantly to treatment with SLC-0111 with reduced viability and thermogenesis when expressing CAIX stimulated by hypoxia (** *p* < 0.01). Error bars indicate one standard deviation.

CAIX and CAXII expression were assessed in several neuroblastoma cell lines under normoxic and hypoxic conditions by immunohistochemical staining (Figure 2, Supplementary Figure S1). SH-EP cells showed strong CAIX expression under normoxic conditions, which even increased under hypoxia. In contrast to SH-EP cells, LAN1 cells did not express CAIX under normoxic conditions and expression of CAIX could not be induced by hypoxia treatment. In SH-SY5Y cells, which did not express CAIX under normoxia, CAIX expression could be induced by hypoxia (Figure 2, first column). As the carbonic anhydrase inhibitor SLC-011 has a high affinity for both CAIX and CAXII [29], CAXII immunohistochemical staining of cells was performed to rule out a significant impact of CAXII. While SH-EP and LAN1 cells showed a slight expression of CAXII under normoxia, which remained unchanged after exposure to hypoxia, SH-SY5Y cells showed a slight expression under normoxia that increased with hypoxia treatment (Supplementary Figure S1). These three cell lines were chosen for further evaluation as they display a variety of different possible CAIX expression patterns.

### 2.3. Neuroblastoma Cell Viability and Thermogenesis under the Inhibition of CAIX and CAXII with SLC-0111

Analysis of the inhibitory effect of SLC-0111 on neuroblastoma cells was performed using a viability assay (Figure 2, second column). Corresponding with their respective CAIX expression patterns, viability of SH-EP cells was significantly reduced upon treatment of SLC-0111 compared with the control under normoxic and hypoxic conditions. The inhibitor did not show an inhibitory effect on LAN1 cells that did not express CAIX. The putatively proliferative effect of SLC-011 in the absence of CAIX/CAXII expression warrants further research. Most interestingly, the effect of CAIX inhibition in SH-SY5Y cells can be induced with the induction of CAIX expression under hypoxia.

Furthermore, we investigated if the inhibitory effect of SLC-0111 can be measured by using isothermal microcalorimetry to make possible rapid treatment evaluation. As hypothesized, heat flow per second (µW/s) recorded a decreased thermogenesis of SLC-0111-treated cells in the presence of CAIX, namely SH-EP cells under normoxic and even more so under hypoxic conditions, in addition to SH-SY5Y cells under hypoxic conditions (Figure 2, third and fourth columns). This effect can be quantified when analyzing total released heat over the observation period (µJ) (Figure 2, fifth column). Thermogenesis in cells treated with SLC-0111 is significantly reduced compared with untreated samples in all three cases. On the other hand, cells that do not express relevant amounts of CAIX, such as LAN1 under normoxia or hypoxia and SH-SY5Y under normoxia, show slight differences in heat flow in addition to the total heat between treated and untreated cells, but are not significant. These slight changes are most likely a result of slight CAIX expression of SH-SY5Y cells (normoxia) and slight CAXII expression in LAN1 (normoxia and hypoxia) and SH-SY5Y (normoxia) cells.

### 2.4. Expression of CAIX and CAXII in Neuroblastoma Patient Samples

**Figure 3:**
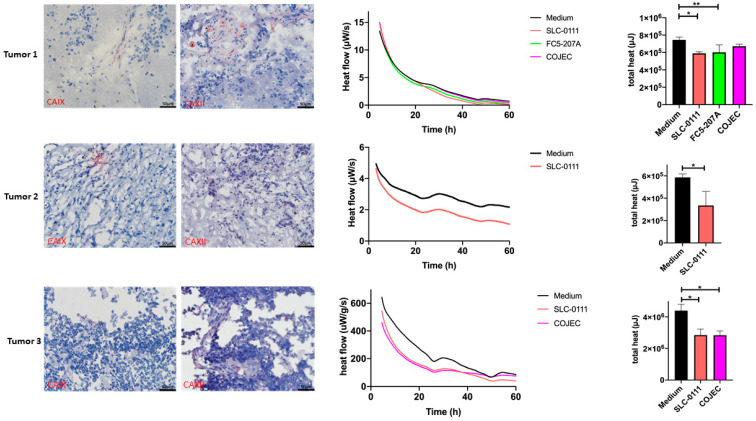
CAIX and CAXII expression of neuroblastoma and monitoring of its inhibition in the organotypic slice culture. Expression and SLC-0111 treatment experiments are shown of three patient tumors in each row. The first two columns show immunohistochemical stainings, and the second two columns depict microcalorimetric measurements of heat flow over time (µW/s) and total heat (µJ). All three tumors show expression of either CAIX or CAXII to varying extents. Treatment of tumor slice 1 was more effective using SLC-0111 and the CAIX inhibitor FC5-207A compared to COJEC (* *p* < 0.05; ** *p* < 0.01), as measured by microcalorimetry. There was only enough tumor material to attempt one treatment for tumor slice 2. Significantly reduced thermogenesis was observed under treatment with SLC-0111 (* *p* < 0.05). Tumor slice 3 was treated with SLC-0111 in addition to COJEC, which reduced thermogenesis by a similar extent (* *p* < 0.05). Error bars indicate one standard deviation.

**Figure 4:**
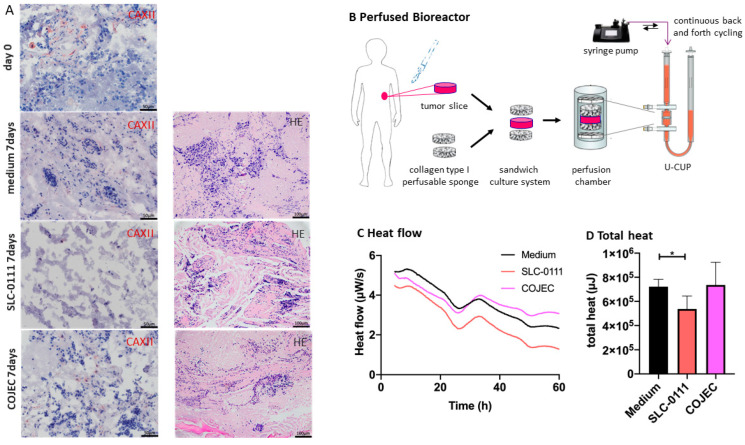
CAXII expression and monitoring of its inhibition in a perfusion-based bioreactor system. (**A**) CAXII expression of original tumor tissue (harvested immediately after resection, row 1). Tissue 7 days after culturing in the perfused bioreactor without treatment showing an intact tumor structure (row 2). Tumor tissue treated with 100 µM SCL-0111 (row 3) and COJEC (row 4), respectively, for 7 days in the perfused bioreactor showing different degrees of tumor structure and tumor cell degradation. In the second column, HE staining of tumor slice after 7 days in medium, SLC-0111 and COJEC are shown. (**B**) Schematic view of bioreactor set up: after tumor resection, tumor slices are placed between two collagen sponges in a sandwich-like fashion into the perfusion chamber and cultured with a continuously cycling medium with or without treatment. (**C**,**D**) Heat flow over time (µW/s) and total heat (µJ) (corresponding to time of heat flow observation) are significantly reduced under treatment with SLC0111 (* *p* < 0.05), but not with COJEC. Error bars indicate one standard deviation.

After confirming the inhibitory effect of SLC-0111 on neuroblastoma cells in vitro, the next step was to transfer this treatment to patient-derived slice cultures. Neuroblastoma is a rare disease and oftentimes a biopsy is taken only at the time of diagnosis. Mostly, tissue is needed for diagnostic purposes. This results in a scarcity of patient tumor tissue that can be used for research. Nevertheless, we included three consecutive neuroblastoma patients in this study. Characteristics of patients and tumors are summarized in Table 1. Tumor tissue was taken directly from the operating room and prepared in part for immunohistochemical analysis (Figure 3, first column), and in part for microcalorimetric measurements (Figure 3, second and third column). For patient 1, additional tumor material was available and was used for culture in a 3D perfusion-based bioreactor system (Figure 4). For each tumor sample, immunohistochemical staining was performed and showed heterogeneous expressions of CAIX and CAXII. Sample 1 strongly expressed CAXII with only slight expression of CAIX. Tumor tissue from patient 2 showed strong expression of CAIX in parts of the tumor and only slight expression of CAXII. Tumor tissue from patient 3 showed expression of CAXII with only slight expression of CAIX.

### 2.5. Treatment Response Evaluation of Treatment with SLC-0111 and COJEC in the Patient Slice Culture Model Using Microcalorimetry

Organotypic slice cultures were treated with either SLC-0111 or medium as control, depending on the amount of available tissue treatment arms with COJEC [30] and the CAIX-specific inhibitor FC5-207A that were included in the experiment. Rapid COJEC refers to a protocol which is used during the induction phase, and consists of alternating sequences of a combination of vincristine, carboplatin/cisplatin and etoposide. We have previously demonstrated the inhibitory effect of FC5-207A on neuroblastoma cells in vitro [24]. The response to treatment was measured by isothermal microcalorimetry by measuring the heat flow per second (µW/s) in addition to the total released heat over the observation period (µJ). In Figure 3, tumor sample 1 reacted with a reduction in thermogenesis in all three treatment groups compared to culture in medium alone. However, only changes for treatments with SLC-0111 and FC5-207A were significant. The standard chemotherapeutic combination therapy COJEC was not as effective. For tumor sample 2, only two groups (SLC-0111 and control) could be examined and showed a significant inhibitory effect which resulted in reduced thermogenesis in the tissue treated with SLC-0111. Tumor sample 3 was treated with SLC-0111 and COJEC. Here, both treatments resulted in a similar and significantly effective reductions in thermogenesis compared with the control samples.

One major advantage of using isothermal microcalorimetry for evaluation of treatment response is the fact that the effect can be evaluated using an organotypic tumor slice culture. Another major advantage is the short time interval of 24–48 h in which the treatment response can be prognosticated.

### 2.6. Increase in Evaluation Window by Tumor Slice Culturing in a Perfused Bioreactor in Combination with Microcalorimetric Treatment Response Evaluation

The short observation time frame which is needed for evaluation by isothermal microcalorimetry is also one of the limitations of using this method. Especially when evaluating the effect of novel therapeutics that influence the tumor’s microenvironment, an extended observation period would present an even greater advantage. Therefore, we established a perfusion-based approach for neuroblastoma tissue in a 3D bioreactor system. Using this system, we maintain an excellent tissue quality with intact tumor cells and stromal structure in neuroblastoma tumors for up to 10 days (Huo et al., submitted). In the current experiment, tumor tissue from sample 1 was treated in a sandwich-like assembly and cultured under perfusion flow in a continuous cycling of medium (Figure 4). The tumor slice was treated with SLC-0111, COJEC and medium alone. After 7 days, tumor tissue was harvested and partially used for immunohistochemical analysis and for isothermal microcalorimetry. While tumor structure was preserved in the medium alone samples, CAXII expression had decreased. In the COJEC-treated group tumor structure was severely disturbed, and treatment with SLC-0111 resulted in an almost complete depletion of tumor cells after 7 days. Microcalorimetry was initiated upon removal of the tumor slices from the bioreactor. Thermogenesis mirrored the findings of the histological observations and was significantly reduced in SLC-0111-treated samples compared to medium control, but not in the COJEC group. Overall values for heat flow and total heat were reduced after 7 days compared with the heat flow and total heat of fresh samples. Nevertheless, treatment response can still be assessed after 7 days in the perfused tumor slice culture.

## 3. Discussion

Here we show the successful inhibition of neuroblastoma cells and patients’ tumors using the CAIX/CAXII inhibitor SLC-0111, which is currently in phase Ib/II clinical trials. We demonstrate the novel use of a patient slice-culture perfusion-based 3D neuroblastoma model in combination with isothermal microcalorimetry for rapid drug response assessment. In the following sections we will discuss neuroblastoma reasons for targeting hypoxia-induced tumor-specific processes, the possibilities of carbonic anhydrase-targeted therapy, advantages of perfusion-based, refined 3D culturing systems and the potential of microcalorimetry for individualized rapid drug response assessment. The need for developing not only new treatment options for advanced neuroblastoma but also for improving preclinical testing strategies for potential therapeutics is obvious. Here we propose a possible strategy exemplified for treatment with the novel inhibitor SLC-0111.

### 3.1. Tumor Cell Heterogeneity and the Hypoxic Microenvironment

Neuroblastoma tumors, as well as stable neuroblastoma cell lines, are prone to display great inter- and intratumor heterogeneity. The heterogeneity is especially pronounced in this tumor entity that arises from developing neuroblasts, which in themselves can be found in various stages of differentiation. A classic example is the cell line SH-SY5Y that can be differentiated from ever-changing neuroblasts into mature neurons [31]. Our previous research has shown that even simple factors such as, e.g., cell density, can influence vital prognostic factors [28]. Moreover, hypoxia can significantly increase known adverse factors in neuroblastoma such as NMYC, CXCR4, PGK1 and AQP1, amongst others, leading to tumor progression and increased metastases [25,26,27,28]. Neuroblastoma cells are characterized by changing biological behavior; a heterogeneous tumor microenvironment with stroma-rich or stroma-poor features; the presence or absence of immunomarkers such as GD2; adverse factors such as NMYC; or adhesion factors such as NCAM. Hypoxic changes of the microenvironment, however, are common to most advanced tumors and become even more prevalent in fast-growing tumors, making targeting of hypoxic factors a sensible approach. Adaptation of cancer cells to the hypoxic microenvironment is regulated through physiological responses to hypoxia that are mediated by hypoxia-inducible factors, namely HIF-1α and HIF-2α. CAIX is highly upregulated in tumors under the effect of HIF-1 [29,32,33]. HIF-1 promotes the expression of genes which regulate the carbon metabolism cycle, resulting in a shift to anaerobic glycolysis and a better survivability of tumor cells under hypoxia [34]. Lactate (produced by anaerobic glycolysis) in combination with the insufficient neovascularization and high metabolic turnover of malignant tumors result in an accumulation of H^+^ ions and, inevitably, to intracellular acidification [35,36]. In order to cope with both acidic and hypoxic stress, different molecular mechanisms are established by the tumor. Known mechanisms include isoforms of carbonic anhydrase or channels that convert or transport either HCO_3^−^_ or H^+^ [32]. The described imbalance in oxygen and acid balance were known about since the early 1930s, when Nobel prize winner Otto Warburg defined them in his Warburg hypothesis [37]. Efforts to target hypoxia-related factors of different tumor cell attributes or tumor compartments have been made since then [29]. Similarly to most carbonic anhydrases, CAIX catalyzes the reversible hydration of carbon dioxide to bicarbonate (CO_2_ + H_2_O ↔ HCO_3^−^_ + H^+^). After extracellular catalysis by carbonic anhydrase, HCO_3^−^_ is transported to the intracellular space with the help of bicarbonate transporters (NBC). Intracellular HCO_3^−^_ then acts as a buffer and ensures a normal to alkaline pH. By transporting the bicarbonate away from the extracellular space, this mechanism also contributes to an acidic extracellular environment [32,38]. Consequently, CAIX ensures an intracellular pH (pHi) that is favorable for cell viability, and also contributes to an acidic extracellular pH (pHe) which promotes the metastatic potential of the cancer [32,39]. Therefore, the inhibition of CAIX has been considered as an anticancer strategy in hypoxic solid tumors [21].

### 3.2. Inhibition with CAIX Inhibitors

Two of the 16 known human CA isoforms, CAIX and CAXII, are predominantly found in tumor cells and show a rather limited expression in normal cells [29]. Evidence for the roles of the specific carbonic anhydrase isoenzymes is accumulating as more research is published on this field. In vivo and in vitro studies have shown that CAIX supports cell survival in an acidic environment, leading to a higher risk of disease progression and metastasis development, and have moreover shown that CAIX can be used as a predictor of survival [40,41,42]. On a molecular level, Ciccone et al. demonstrated an increase in pERK1/2 in their models which were under the effect of SLC-0111. pERK1/2 is known as a typical cell survival marker which activates p53, leading to apoptosis of the cell [43]. Therefore, CAIX can be considered as a potential but crucial target for new anticancer therapies. Due to the role of CAIX in cancer development, a stronger effect of CAIX inhibitors can be expected in cancer cells with high invasive potential [43]. Furthermore, SLC-0111 seems to be a selective inhibitor for CAIX and CAXII which further favors the use of this compound as a new and selective anticancer therapy [29,44]. Other authors found a compensatory increase in CAXII when they suppressed CAIX [40]. The first human phase 1 study to determine a starting dose was published in 2020 and showed promising data [36]. A phase Ib/II clinical trial is currently ongoing. However, further investigation is needed to define the clinical usage of SLC-0111 as an anticancer treatment. Other authors have specifically tested the use of CAIX inhibitors in combination with conventional chemotherapeutics and received some promising results [45,46,47]. Overall, the existing data are auspicious regarding the use of SLC-0111 as an anticancer treatment. In comparison to treatment with the chemotherapeutic induction therapy COJEC, we observed a high effectiveness of SCL-0111 in the presented tumor tissues.

### 3.3. Culturing Conditions

Recently, several 3D in vitro models for pre-clinical drug assessment have emerged. There are, for example, systems using multicellular tumor spheroids (MCTSs) that can be composed of single or multiple cell types, with or without ECM support. ECM support can be provided, e.g., in the form of scaffolds. Tissue-derived tumor spheres (TDTSs) isolated from patient tumor cells are used in other models, which are more often derived from metastatic bone marrow aspirate cultured in Matrigel [48,49,50,51]. These have successfully been applied to neuroblastoma.

A further step has been made by using patient-derived tumor organoids (PDTOs) generated from embryonic stem cells (ECS) and expressing strong phenotypic and genetic similarities with the original tumors [18]. This system, however, has mostly been applied for tumors with an epithelial background and still is undeveloped for tumors without an epithelial background, such as neuroblastoma [17].

Due to current clinical study practices, patient tumor tissue at the time of diagnosis of neuroblastoma is scarce. If at all, a biopsy is taken which is needed for inclusion into clinical trials. As a result of improvements in chemo- and immunotherapeutic approaches, excision biopsies of suspected neuroblastomas are rare. At the same time, it becomes apparent that investigation of a tumor in its natural structure is vital. Both the extracellular matrix (ECM) and the influence of the immune environment on the cancer cells are of great interest. Several groups are experimenting with using 3D scaffolds, mostly collagen-based or (bio)printed structures. Our group has recently been able to establish a perfusion-based mono-cell type 3D neuroblastoma scaffold (Huo et al., submitted).

One way to facilitate advancement from 2D to 3D culture has been through the introduction of a perfusion-based bioreactor culturing system. Previous studies on tumors of epithelial origin have yielded excellent results using organotypic slice cultures of colorectal and breast tumors [16,52,53]. In this study we describe the use of 3D perfusion-based organotypic slice cultures for neuroblastoma for the first time. Continuous perfusion of the specimen that is embedded sandwich-like in a collagen scaffold increases tumor slice access of medium components such as nutrients, improves oxygen supply and removal of CO_2_, as well as delivery of the drug to cancer cells. The collagen scaffolds help stabilize and preserve the tissue structure while not hindering substrate supply. While 2D or 3D culturing options allow drug response assessment by using conventional assays such as cell proliferation or viability assays, drug response assessment of organotypic slice cultures has been limited to histological evaluation, RNA or protein analysis.

### 3.4. Isothermal Microcalorimetry for Treatment Response Assessment

Individualized therapeutic approaches need to be evaluated in the entire intact tumor structure of each child’s individual tumor, not in a single cell type culture or in 2D models or in 3D models. Thus, new systems have to be evaluated to assess drug response. We have previously demonstrated the effectiveness of evaluating novel therapeutic approaches in rare childhood tumors using isothermal microcalorimetry of organotypic slice cultures [19]. More recently, we have been able to link a higher metastatic potential of tumor cells to biophysical properties, such as decreased adherence and increased thermogenesis [54]. Isothermal microcalorimetry can also be of advantage in evaluating thermogenesis of neuroblastoma cells under stimulation with different nutrient supplies [55].

One major advantage of using isothermal microcalorimetry for evaluation of treatment response is the fact that the effect can be evaluated using an organotypic tumor slice culture. In our current study we show that the perfused control tissue is still viable after 7 days. Clear differences in treatment groups can be observed and measured by microcalorimetric evaluation. This prolonged evaluation period opens the possibilities of evaluating more complex treatment regimens of applying immune-modifying treatments, for which longer time periods are necessary.

Inversely, another major advantage is the short time interval of 24–48 h, after which the treatment response can be prognosticated.

The use of tumor treatment in the perfusion-based bioreactor followed by microcalorimetric evaluation allows longer evaluation periods followed by rapid response assessment. In this way our experimental setup allows maximum use of scarce neuroblastoma tissue and evaluation of patient-tailored treatment protocols.

## 4. Materials and Methods

### 4.1. Tissue Microarray (TMA)

Tissue samples were fixed in 4% buffered formalin, paraffin-embedded and used for TMA construction as previously described [56]. Briefly, hematoxylin-eosin-stained sections were made from selected primary tumor blocks (donor blocks) to define representative tumor regions. Tissue cylinders (0.6 mm in diameter) were then punched from that region of the donor block using a home-made semi-automated tissue arrayer. Sections of 3 μm were made by use of the Paraffin Sectioning Aid System (Instrumentics, Hackensack, NJ, USA). For the analysis, only patients whose tissue was present on the TMA were included (Figure 1). All patient samples were analyzed by the Department of Pathology of the University Hospital Basel. The analysis was limited to primary neuroblastoma samples of 34 patients for CAIX staining and 30 patients for CAXII staining; additionally, 16 samples for CAIX staining and 8 patients for CAXII staining with healthy adrenal glands were used. Only for these samples the sample quality was sufficient. Information on staging and outcome were not available for any patient. The use of the TMA and corresponding patient information were approved by the appropriate ethics committee (Ethikkommission Nordwest- und Zentralschweiz EKNZ 2015-263).

### 4.2. Patient Tumor Samples

Patient-derived tumor samples were taken from the resected specimen immediately after surgery and used for either cryo-conservation, treatment experiments in static in vitro culture, perfusion bioreactor experiments or isothermal microcalorimetry. Written consent was obtained prior to the operation and use of human tissue was conducted in accordance with ethics approval (EKNZ 2015-263).

### 4.3. Cell Culture

Neuroblastoma SH-SY5Y (ECACC/Sigma-Aldrich, Munich, Germany), SH-EP and LAN1 cells (ECACC, kindly provided by A. Muhlethaler, CHUV) were cultivated in RPMI and DMEM media containing 10% FCS. If possible, aliquots of early passages (4–6) after purchase were used for all experiments. All cells were cultured in a humidified atmosphere at 37 °C either in air with 5% CO_2_ under normoxic conditions, or with 5% CO_2_/1–5% O_2_ balanced with N_2_ under hypoxic conditions. The CAIX/CAXII inhibitor SLC-0111 [36,45], 4-[(4-fluorophenyl) carbamoyl] amino-benzene sulfonamide, was kindly provided by C. Supuran, and was added to the medium to a final concentration of 100 µM for all treatment experiments. CAIX inhibitor FC5-207A [24] was used at final concentrations of 500 µM.

### 4.4. Immunohistochemical Staining

TMA was stained by the Department of Pathology of the University Hospital Basel according to the validated protocol using the appropriate CAIX antibody (Ventana Clone EP161 Rabbit, Roche diagnostics, Rotkreuz, Switzerland). The CAXII staining was performed using the primary antibody CA12 Rabbit PolyAb (Catalog No. 15180-1-AP, Proteintech Europe, Manchester, UK) at a dilution of 1:300. The scoring was performed according to a modification of the scaling system used for clinical scoring of molecular markers, combining a score for area and intensity by two independent blinded examiners, a pathologist (EB), an IHC experienced surgeon (SJG) and a scientist (ZH).

For immunohistochemical staining, 5 × 10^4^ cells per chamber were seeded in chamber slides (Thermofisher/Sigma Aldrich, Munich, Germany). The cells were incubated for 24 h either under normoxic or hypoxic conditions (5% CO_2_, 1% O_2_ in balance with N_2_ at 37 °C, respectively). After 24 h cells were fixated with 4% PFA followed by immunohistochemical staining using the HRP-AEC-System from R&D Systems (Minneapolis, MN, USA). For patient tumor samples, tissues were cryo- or paraffin-embedded after treatment in the perfusion bioreactor slice culture and cut to a thickness of 5 µm. The CAIX staining was performed using the primary antibody M75 (BioScience Slovakia, Bratislava, Slovak republic) at a dilution of 1:200. The CAXII staining was performed using the primary antibody CA12 Rabbit PolyAb (Catalog No. 15180-1-AP, Proteintech Europe, Manchester, UK) at a dilution of 1:300. Control sections were incubated with antibody diluent (DAKO, Glostrup, Denmark) without primary antibody at 4 °C overnight and then treated as other samples. For counterstaining, Mayer’s hematoxylin solution (Spitalpharmazie, Basel, Switzerland) was applied. A BX43 Olympus Microscope was used for analysis and images were recorded using Cell Sense Software at a standard magnification of 20×.

### 4.5. Cell Proliferation Assay

Cells were seeded in two culture dishes and primed in either normoxic or hypoxic (5% CO_2_, 2% O_2_ in balance with N_2_ at 37 °C, respectively) conditions for 48 h. After this period, cells were seeded at 10,000 cells/well in 96-well plates separately and settled overnight. The inhibitor SLC-0111 was added to the cells in concentrations of 0 µM and 100 µM. Cells were further cultured under hypoxic or normoxic conditions. The MTT assay (CellTiter 96 Aqueous One Solution Cell Proliferation Assay, Promega) was carried out in accordance with the manufacturer’s protocol at 24 h. Absorbance was measured at 490 nm with a Synergy Hybrid H4 Reader (BioTek, Sursee, Switzerland). Each experiment was performed at least six times.

### 4.6. Isothermal Microcalorimetry

For microcalorimetric measurements a 48-channel isothermal microcalorimeter (calScreener, Symcel AB, Stockholm, Sweden) was used as previously described [57]. Tumor cells were seeded into vials in their respective media and incubated for 6 h to attach. Alternatively, tissue slice culture pieces were prepared using scalpel dissection under the binocular in a standardized manner. Tissue slices were cultured in TUM medium [58,59] at 37 °C. Specific experimental inhibitors (CAIX inhibitors (SLC-0111 at 100 µM, FC5-207A at 500 μM) and standard induction chemotherapy COJEC according to the high-risk protocol of the SIOPEN study were applied [30,60,61] including vincristine, cisplatin and etoposide. Control samples containing TUM medium alone were included (n = 4 per group).

The vials were then sealed and inserted in the well-plate microcalorimeter according to manufacturers’ instructions. One position on the plate was loaded with an inert sample, which was used as a reference. For optimal performance, multiple separate reference vessels were included. Each reference vessel was filled with an inert sample (medium only), which was used as a thermal reference. Following that, thermal equilibration measurements were recorded with the thermostat set at 37 °C. The microcalorimeter data were sampled at a frequency of 1 data point every 60 s over >250 h until the metabolic heat signal returned to baseline. Data were stored by Symcel calView software and exported as a CSV file that could be edited in commonly used spreadsheet software. This assay was performed in triplicate and repeated. Data were analyzed using GraphPad Prism 8.4 software. For testing of significance, a two-sided *t*-test or analysis of variance followed by Dunnett’s post-hoc test was used. *p* values less than 0.05 were defined as significant. The error bars in all bar plots represent one standard deviation.

### 4.7. Perfusion-Based Bioreactor Culture

Four tumor sections were placed between two discs in a sandwich-like configuration (Patent Nr. WO2015181185A1) into the perfusion bioreactor filled with medium that is continuously pumped back and forth through the tissue sandwich. The scaffold discs (diameter 8 mm, height 3 mm), made from a porous water-insoluble partial hydrochloric acid salt of purified bovine corium collagen sponge, known as Ultrafoam Collagen Hemostat (Avitene, Bard), were soaked in culture medium for 1 h at 37 °C. The sandwich was assembled within a ring-shaped silicon holder closed on top and bottom by two ETFE nylon meshes (Fluorotex Sefar, 09-590/47). The scaffold assembly was then placed into a previously described perfusion bioreactor [62] (currently distributed as U-CUP Bioreactor Cellec Biotek AG) and perfused with 8 mL of TUM medium [58,59]. SLC-0111 and COJEC treatment reagents were added to final concentrations corresponding to the ones used for isothermal microcalorimetry. Perfusion superficial velocity was set at 100 μm/s, as previously described [63,64,65]. Treatment was renewed every 48 h during the experiment. After 7 days of culturing tissues were removed and cryo-conserved or paraffin-embedded for further analysis.

## 5. Conclusions

The need for developing and improving advanced preclinical testing strategies for potential therapeutics pertaining to neuroblastoma is enormous. Here we propose the use of a new patient-derived 3D slice-culture perfusion-based 3D model in combination with rapid treatment evaluation using isothermal microcalorimetry exemplified for treatment with the novel carbonic anhydrase inhibitor SLC-0111. This approach can facilitate individualization and improvements in treatment strategies for each patient.

## Figures and Tables

**Table 1:** Neuroblastoma patient characteristics.

	Patient 1	Patient 2	Patient 3
Classification	stage I	stage III	stage IV high risk
Location	adrenal gland	thoracical	adrenal gland
Metastases	negative	negative	ubiquitary metastases (MIBG scintigraphy)
Histology	poorly differentiated, stroma-poor	poorly differentiated, stroma-poor	undifferentiated, stroma-poor, high mitosis rate
NMYC Expression	negative	negative	negative

## Data Availability

Not applicable.

## Supplementary Materials

The following supporting information can be downloaded at: https://www.mdpi.com/article/10.3390/ijms23063128/s1.

## References

[1] Cheung N.K., Dyer M.A. (2013). Neuroblastoma: Developmental biology, cancer genomics and immunotherapy. Nat. Rev. Cancer.

[2] Liang S.W., Chen G., Luo Y.G., Chen P., Gu J.H., Xu Q.Q., Dang Y.W., Qin L.T., Lu H.P., Huang W.T. (2020). Nomogram for predicting overall survival in children with neuroblastoma based on SEER database. Ann. Surg. Treat. Res..

[3] Kimura S., Sekiguchi M., Watanabe K., Hiwatarai M., Seki M., Yoshida K., Isobe T., Shiozawa Y., Suzuki H., Hoshino N. (2021). Association of high-risk neuroblastoma classification based on expression profiles with differentiation and metabolism. PLoS ONE.

[4] Davidoff A.M. (2012). Neuroblastoma. Semin. Pediatr. Surg..

[5] Gatta G., Botta L., Rossi S., Aareleid T., Bielska-Lasota M., Clavel J., Dimitrova N., Jakab Z., Kaatsch P., Lacour B. (2014). Childhood cancer survival in Europe 1999–2007: Results of EUROCARE-5—A population-based study. Lancet Oncol..

[6] Tolbert V.P., Matthay K.K. (2018). Neuroblastoma: Clinical and biological approach to risk stratification and treatment. Cell Tissue Res..

[7] Applebaum M.A., Desai A.V., Glade Bender J.L., Cohn S.L. (2017). Emerging and investigational therapies for neuroblastoma. Expert Opin. Orphan Drugs.

[8] Adamson P.C. (2017). Challenges in drug development for children. Clin. Adv. Hematol. Oncol..

[9] Boeva V., Louis-Brennetot C., Peltier A., Durand S., Pierre-Eugene C., Raynal V., Etchevers H.C., Thomas S., Lermine A., Daudigeos-Dubus E. (2017). Heterogeneity of neuroblastoma cell identity defined by transcriptional circuitries. Nat. Genet..

[10] Peinemann F., van Dalen E.C., Enk H., Berthold F. (2017). Retinoic acid postconsolidation therapy for high-risk neuroblastoma patients treated with autologous haematopoietic stem cell transplantation. Cochrane Database Syst. Rev..

[11] Hay M., Thomas D.W., Craighead J.L., Economides C., Rosenthal J. (2014). Clinical development success rates for investigational drugs. Nat. Biotechnol..

[12] Martin I., Wendt D., Heberer M. (2004). The role of bioreactors in tissue engineering. Trends Biotechnol..

[13] Cerino G., Gaudiello E., Grussenmeyer T., Melly L., Massai D., Banfi A., Martin I., Eckstein F., Grapow M., Marsano A. (2016). Three dimensional multi-cellular muscle-like tissue engineering in perfusion-based bioreactors. Biotechnol. Bioeng..

[14] Bourgine P.E., Klein T., Paczulla A.M., Shimizu T., Kunz L., Kokkaliaris K.D., Coutu D.L., Lengerke C., Skoda R., Schroeder T. (2018). In vitro biomimetic engineering of a human hematopoietic niche with functional properties. Proc. Natl. Acad. Sci. USA.

[15] Muraro M.G., Muenst S., Mele V., Quagliata L., Iezzi G., Tzankov A., Weber W.P., Spagnoli G.C., Soysal S.D. (2017). Ex-vivo assessment of drug response on breast cancer primary tissue with preserved microenvironments. Oncoimmunology.

[16] Manfredonia C., Muraro M.G., Hirt C., Mele V., Governa V., Papadimitropoulos A., Daster S., Soysal S.D., Droeser R.A., Mechera R. (2019). Maintenance of Primary Human Colorectal Cancer Microenvironment Using a Perfusion Bioreactor-Based 3D Culture System. Adv. Biosyst..

[17] Bleijs M., van de Wetering M., Clevers H., Drost J. (2019). Xenograft and organoid model systems in cancer research. EMBO J..

[18] Clevers H. (2016). Modeling Development and Disease with Organoids. Cell.

[19] Gros S.J., Holland-Cunz S.G., Supuran C.T., Braissant O. (2019). Personalized Treatment Response Assessment for Rare Childhood Tumors Using Microcalorimetry-Exemplified by Use of Carbonic Anhydrase IX and Aquaporin 1 Inhibitors. Int. J. Mol. Sci..

[20] Semenza G.L. (2012). Hypoxia-inducible factors: Mediators of cancer progression and targets for cancer therapy. Trends Pharmacol. Sci..

[21] Chafe S.C., Vizeacoumar F.S., Venkateswaran G., Nemirovsky O., Awrey S., Brown W.S., McDonald P.C., Carta F., Metcalfe A., Karasinska J.M. (2021). Genome-wide synthetic lethal screen unveils novel CAIX-NFS1/xCT axis as a targetable vulnerability in hypoxic solid tumors. Sci. Adv..

[22] Lou Y., McDonald P.C., Oloumi A., Chia S., Ostlund C., Ahmadi A., Kyle A., Auf dem Keller U., Leung S., Huntsman D. (2011). Targeting tumor hypoxia: Suppression of breast tumor growth and metastasis by novel carbonic anhydrase IX inhibitors. Cancer Res..

[23] Fiaschi T., Giannoni E., Taddei M.L., Cirri P., Marini A., Pintus G., Nativi C., Richichi B., Scozzafava A., Carta F. (2013). Carbonic anhydrase IX from cancer-associated fibroblasts drives epithelial-mesenchymal transition in prostate carcinoma cells. Cell Cycle.

[24] Ameis H.M., Drenckhan A., Freytag M., Izbicki J.R., Supuran C.T., Reinshagen K., Holland-Cunz S., Gros S.J. (2016). Carbonic anhydrase IX correlates with survival and is a potential therapeutic target for neuroblastoma. J. Enzym. Inhib. Med. Chem..

[25] Ameis H.M., Drenckhan A., Freytag M., Izbicki J.R., Supuran C.T., Reinshagen K., Holland-Cunz S., Gros S.J. (2016). Influence of hypoxia-dependent factors on the progression of neuroblastoma. Pediatric Surg. Int..

[26] Ameis H.M., Drenckhan A., von Loga K., Escherich G., Wenke K., Izbicki J.R., Reinshagen K., Gros S.J. (2013). PGK1 as predictor of CXCR4 expression, bone marrow metastases and survival in neuroblastoma. PLoS ONE.

[27] Huo Z., Lomora M., Kym U., Palivan C., Holland-Cunz S.G., Gros S.J. (2021). AQP1 Is Up-Regulated by Hypoxia and Leads to Increased Cell Water Permeability, Motility, and Migration in Neuroblastoma. Front. Cell. Dev. Biol..

[28] Pini N., Huo Z., Kym U., Holland-Cunz S., Gros S.J. (2021). AQP1-Driven Migration Is Independent of Other Known Adverse Factors but Requires a Hypoxic Undifferentiated Cell Profile in Neuroblastoma. Children.

[29] Supuran C.T. (2020). Experimental Carbonic Anhydrase Inhibitors for the Treatment of Hypoxic Tumors. J. Exp. Pharm..

[30] Ladenstein R., Potschger U., Pearson A.D.J., Brock P., Luksch R., Castel V., Yaniv I., Papadakis V., Laureys G., Malis J. (2017). Busulfan and melphalan versus carboplatin, etoposide, and melphalan as high-dose chemotherapy for high-risk neuroblastoma (HR-NBL1/SIOPEN): An international, randomised, multi-arm, open-label, phase 3 trial. Lancet Oncol..

[31] Shipley M.M., Mangold C.A., Szpara M.L. (2016). Differentiation of the SH-SY5Y Human Neuroblastoma Cell Line. J. Vis. Exp. JoVE.

[32] Neri D., Supuran C.T. (2011). Interfering with pH regulation in tumours as a therapeutic strategy. Nat. Rev. Drug Discov..

[33] Vaupel P., Mayer A. (2016). Tumor Hypoxia: Causative Mechanisms, Microregional Heterogeneities, and the Role of Tissue-Based Hypoxia Markers. Adv. Exp. Med. Biol..

[34] Xie H., Simon M.C. (2017). Oxygen availability and metabolic reprogramming in cancer. J. Biol. Chem..

[35] Corbet C., Feron O. (2017). Tumour acidosis: From the passenger to the driver’s seat. Nat. Rev. Cancer.

[36] McDonald P.C., Chia S., Bedard P.L., Chu Q., Lyle M., Tang L., Singh M., Zhang Z., Supuran C.T., Renouf D.J. (2020). A Phase 1 Study of SLC-0111, a Novel Inhibitor of Carbonic Anhydrase IX, in Patients With Advanced Solid Tumors. Am. J. Clin. Oncol..

[37] Warburg O. (1956). On the origin of cancer cells. Science.

[38] Pastorekova S., Gillies R.J. (2019). The role of carbonic anhydrase IX in cancer development: Links to hypoxia, acidosis, and beyond. Cancer Metastasis Rev..

[39] Huber V., De Milito A., Harguindey S., Reshkin S.J., Wahl M.L., Rauch C., Chiesi A., Pouysségur J., Gatenby R.A., Rivoltini L. (2010). Proton dynamics in cancer. J. Transl. Med..

[40] Chiche J., Ilc K., Laferriere J., Trottier E., Dayan F., Mazure N.M., Brahimi-Horn M.C., Pouyssegur J. (2009). Hypoxia-inducible carbonic anhydrase IX and XII promote tumor cell growth by counteracting acidosis through the regulation of the intracellular pH. Cancer Res..

[41] van Kuijk S.J., Yaromina A., Houben R., Niemans R., Lambin P., Dubois L.J. (2016). Prognostic Significance of Carbonic Anhydrase IX Expression in Cancer Patients: A Meta-Analysis. Front. Oncol..

[42] Hussain S.A., Ganesan R., Reynolds G., Gross L., Stevens A., Pastorek J., Murray P.G., Perunovic B., Anwar M.S., Billingham L. (2007). Hypoxia-regulated carbonic anhydrase IX expression is associated with poor survival in patients with invasive breast cancer. Br. J. Cancer.

[43] Ciccone V., Filippelli A., Angeli A., Supuran C.T., Morbidelli L. (2020). Pharmacological Inhibition of CA-IX Impairs Tumor Cell Proliferation, Migration and Invasiveness. Int. J. Mol. Sci..

[44] Angeli A., Carta F., Nocentini A., Winum J.Y., Zalubovskis R., Akdemir A., Onnis V., Eldehna W.M., Capasso C., Simone G. (2020). Carbonic Anhydrase Inhibitors Targeting Metabolism and Tumor Microenvironment. Metabolites.

[45] McDonald P.C., Chafe S.C., Brown W.S., Saberi S., Swayampakula M., Venkateswaran G., Nemirovsky O., Gillespie J.A., Karasinska J.M., Kalloger S.E. (2019). Regulation of pH by Carbonic Anhydrase 9 Mediates Survival of Pancreatic Cancer Cells With Activated KRAS in Response to Hypoxia. Gastroenterology.

[46] Petrenko M., Güttler A., Funtan A., Keßler J., Emmerich D., Paschke R., Vordermark D., Bache M. (2021). Combined 3-O-acetylbetulin treatment and carbonic anhydrase IX inhibition results in additive effects on human breast cancer cells. Chem. Biol. Interact..

[47] Ward C., Meehan J., Gray M., Kunkler I.H., Langdon S.P., Argyle D.J. (2018). Carbonic Anhydrase IX (CAIX), Cancer, and Radiation Responsiveness. Metabolites.

[48] Nolan J.C., Frawley T., Tighe J., Soh H., Curtin C., Piskareva O. (2020). Preclinical models for neuroblastoma: Advances and challenges. Cancer Lett..

[49] Sutherland R.M., McCredie J.A., Inch W.R. (1971). Growth of multicell spheroids in tissue culture as a model of nodular carcinomas. J. Natl. Cancer Inst..

[50] Nath S., Devi G.R. (2016). Three-dimensional culture systems in cancer research: Focus on tumor spheroid model. Pharmacol. Ther..

[51] Thole T.M., Toedling J., Sprussel A., Pfeil S., Savelyeva L., Capper D., Messerschmidt C., Beule D., Groeneveld-Krentz S., Eckert C. (2020). Reflection of neuroblastoma intratumor heterogeneity in the new OHC-NB1 disease model. Int. J. Cancer.

[52] Foglietta F., Spagnoli G.C., Muraro M.G., Ballestri M., Guerrini A., Ferroni C., Aluigi A., Sotgiu G., Varchi G. (2018). Anticancer activity of paclitaxel-loaded keratin nanoparticles in two-dimensional and perfused three-dimensional breast cancer models. Int. J. Nanomed..

[53] Hirt C., Papadimitropoulos A., Muraro M.G., Mele V., Panopoulos E., Cremonesi E., Ivanek R., Schultz-Thater E., Droeser R.A., Mengus C. (2015). Bioreactor-engineered cancer tissue-like structures mimic phenotypes, gene expression profiles and drug resistance patterns observed “in vivo”. Biomaterials.

[54] Huo Z., Sa Santos M., Drenckhan A., Holland-Cunz S., Izbicki J.R., Nash M.A., Gros S.J. (2021). Metastatic Esophageal Carcinoma Cells Exhibit Reduced Adhesion Strength and Enhanced Thermogenesis. Cells.

[55] Pini N., Huo Z., Holland-Cunz S., Gros S.J. (2021). Increased Proliferation of Neuroblastoma Cells under Fructose Metabolism Can Be Measured by Isothermal Microcalorimetry. Children.

[56] Schraml P., Bucher C., Bissig H., Nocito A., Haas P., Wilber K., Seelig S., Kononen J., Mihatsch M.J., Dirnhofer S. (2003). Cyclin E overexpression and amplification in human tumours. J. Pathol..

[57] Braissant O., Keiser J., Meister I., Bachmann A., Wirz D., Gopfert B., Bonkat G., Wadso I. (2015). Isothermal microcalorimetry accurately detects bacteria, tumorous microtissues, and parasitic worms in a label-free well-plate assay. Biotechnol. J..

[58] Gros S.J., Dohrmann T., Peldschus K., Schurr P.G., Kaifi J.T., Kalinina T., Reichelt U., Mann O., Strate T.G., Adam G. (2010). Complementary use of fluorescence and magnetic resonance imaging of metastatic esophageal cancer in a novel orthotopic mouse model. Int. J. Cancer.

[59] Gros S.J., Kurschat N., Dohrmann T., Reichelt U., Dancau A.M., Peldschus K., Adam G., Hoffman R.M., Izbicki J.R., Kaifi J.T. (2010). Effective therapeutic targeting of the overexpressed HER-2 receptor in a highly metastatic orthotopic model of esophageal carcinoma. Mol. Cancer Ther..

[60] Ladenstein R., Potschger U., Valteau-Couanet D., Luksch R., Castel V., Ash S., Laureys G., Brock P., Michon J.M., Owens C. (2020). Investigation of the Role of Dinutuximab Beta-Based Immunotherapy in the SIOPEN High-Risk Neuroblastoma 1 Trial (HR-NBL1). Cancers.

[61] Ladenstein R., Potschger U., Valteau-Couanet D., Luksch R., Castel V., Yaniv I., Laureys G., Brock P., Michon J.M., Owens C. (2018). Interleukin 2 with anti-GD2 antibody ch14.18/CHO (dinutuximab beta) in patients with high-risk neuroblastoma (HR-NBL1/SIOPEN): A multicentre, randomised, phase 3 trial. Lancet. Oncol..

[62] Braccini A., Wendt D., Jaquiery C., Jakob M., Heberer M., Kenins L., Wodnar-Filipowicz A., Quarto R., Martin I. (2005). Three-dimensional perfusion culture of human bone marrow cells and generation of osteoinductive grafts. Stem. Cells.

[63] Cioffi M., Kuffer J., Strobel S., Dubini G., Martin I., Wendt D. (2008). Computational evaluation of oxygen and shear stress distributions in 3D perfusion culture systems: Macro-scale and micro-structured models. J. Biomech..

[64] Santoro M., Lamhamedi-Cherradi S.E., Menegaz B.A., Ludwig J.A., Mikos A.G. (2015). Flow perfusion effects on three-dimensional culture and drug sensitivity of Ewing sarcoma. Proc. Natl. Acad. Sci. USA.

[65] Radisic M., Deen W., Langer R., Vunjak-Novakovic G. (2005). Mathematical model of oxygen distribution in engineered cardiac tissue with parallel channel array perfused with culture medium containing oxygen carriers. Am. J. Physiol. Heart Circ. Physiol..

