# Supplementary material for: Perfusion-Based Bioreactor Culture and Isothermal Microcalorimetry for Preclinical Drug Testing with the Carbonic Anhydrase Inhibitor SLC-0111 in Patient-Derived Neuroblastoma
Source: Int J Mol Sci. 2022 Mar 14;23(6):3128. doi: 10.3390/ijms23063128 (PMC8955558; doi:10.3390/ijms23063128)
Supplement: Supplementary file 1 [file ijms-23-03128-s001.zip › ijms-1628012-supplementary.pdf]

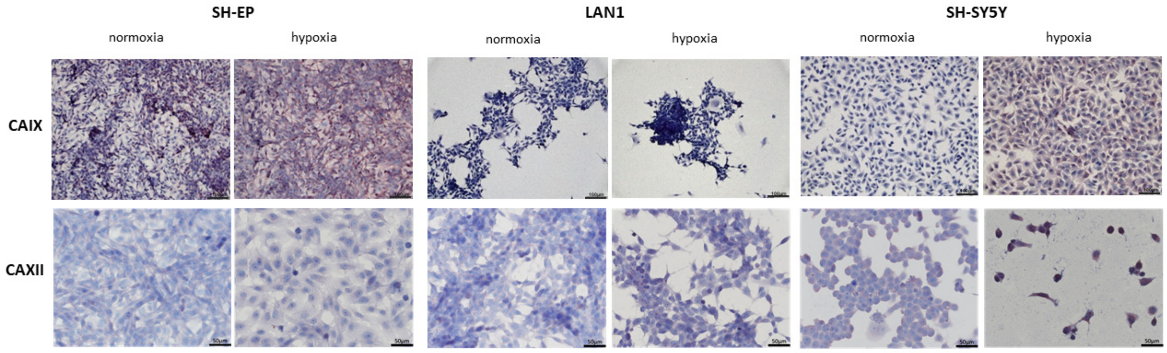

Supplementary Figure S1. Expression of CAIX and CAXII in cell lines. SH-EP cell lines show strong CAIX expression under normoxia which is even stringer under stimulation with hypoxia, while CAXII expression is weak in both conditions. LAN1 cells show no relevant expression of CAIX and only weak CAXII expression. In SH-SY5Y cells CAXII expression in weak under normoxia conditions, while CAIX expression in negative. However, both CAIX and CAXII expression can be stimulated with hypoxia treatment.
